# Effects of an educational compact intervention in self-care – a mixed methods study with postgraduate trainees in primary care

**DOI:** 10.1186/s12875-023-02074-w

**Published:** 2023-06-16

**Authors:** Simon Schwill, Till Johannes Bugaj, Annalena Rentschler, Christoph Nikendei, Joachim Szecsenyi, Katja Krug

**Affiliations:** 1 Department of General Practice and Health Services Research, University of Heidelberg, Heidelberg, Germany; 2 Department of General Internal Medicine and Psychosomatics, University of Heidelberg Medical Hospital, Im Neuenheimer Feld 410, 69120 Heidelberg, Germany

**Keywords:** Self-care, Postgraduate medical education, Family medicine, Educational compact intervention, Behavioral change

## Abstract

**Background:**

Multiple studies indicate that residents in family medicine (FM) are exposed to considerable stress and are particularly affected by burnout syndrome. Aim of the study was to specify the effects of a so-called “compact intervention” (i.e., a short intervention) in self-care on FM residents.

**Methods:**

The authors performed a concurrent and independent mixed-methods study with FM residents on the KWBW Verbundweiterbildung^*PLUS*^© program. FM residents could voluntarily take part in a two-day seminar including 270 min on self-care, which can be regarded as a compact intervention. Study participants completed a questionnaire before (T1) and ten to twelve weeks after the course (T2), with subsequent recruitment to interview. The main outcomes of the quantitative part were to evaluate (I) self-rated change of cognition and (II) change in behavior. The qualitative outcomes were all possible effects of the compact intervention on participants´ competencies as well as all sorts of induced behavioral changes.

**Results:**

From a total of *n* = 307 residents, *n* = 287 FM residents (intervention group: *n* = 212; control group: *n* = 75) participated in the study. At T2, 111 post-intervention questionnaires were completed. 56% rated the intervention to be helpful for their well-being (*n* = 63/111). At T2, there was a significant increase in those willing to act in comparison to T1 (*p* = .01): 36% (*n* = 40/111) had changed their behavior and half of the study participants had passed on competencies to others (*n* = 56/111). From the intervention group, *n* = 17 participants additionally gave an interview. FM residents favored a trustful learning atmosphere, an interactive teaching concept and practical exercises. They described an encouraging stimulus to act and specified behavioral changes.

**Conclusions:**

A compact intervention in self-care could increase well-being, foster competencies and induce behavioral changes, if implemented into a training program with sufficient group cohesiveness. Further studies are required to specify long-term-results.

**Supplementary Information:**

The online version contains supplementary material available at 10.1186/s12875-023-02074-w.

## Introduction.

Studies exploring the prevalence of psychosocial strains among residents in postgraduate medical education frequently report high levels of stress as well as depression and burnout syndrome in virtually all health-care systems [1–3]. Some studies indicate that residents in family medicine (FM) also are exposed to considerable stress [4–7]. FM specialists (family physicians; FPs) are burdened for numerous reasons, such as high workload, various bureaucratic demands and challenging patients e.g. those with multimorbidity [4, 8]. In both Europe and the U.S., a large number of FPs report high levels of stress while being particularly affected by burnout syndrome [5, 9].

Self-care, the process of taking an active role in protecting or improving one's own health, includes caring for one's psychosocial health [4]. To date, several interventions have been described with the goal of psychosocial health promotion for physicians [4]. These can be divided into organizational / structural directed measures and measures of behavior-oriented prevention, which should ideally always be used synergistically [10]. More recently, residency programs in the United States have offered courses in stress management specifically aimed at residents and so-called well-being curricula as components of the training curricula [11]. The impact of these interventions is yet to be described [12]. Furthermore, a tendency can be observed in recent years to increasingly focus on the resilience of physicians in interventions [13]. The methods of well-being and resilience interventions include for example mindfulness-trainings or other stress reduction and meditation or relaxation techniques, or a combination of these features. In addition, trainings that focus on emotional-supportive-coping, elements of psychoeducation or even cognitive and solution-focused counseling are reported. Other interventions that are reported in the literature are self-care workshops, communication and stress management trainings – all of which combine different techniques and are often used for burnout prevention, as well as Balint and support groups [14]. The duration of these interventions ranges from single appointment trainings over a span of two years [13].

In Germany, specialization in FM requires five years of training during clinical work, with mandatory rotations in internal medicine (12 months) and FM (24 months) as well as 24 months of further training in other elective specialist rotations. As of 2008, the KWBW Verbundweiterbildung^*PLUS*^© has been the first German residency training program to offer a seminar curriculum as well as structured mentoring and regional clinical rotations across Baden-Württemberg for FM residents [15, 16]. Participation in the five-year-curriculum is voluntarily and resources are limited. Despite their limited time, educational “compact interventions” on a wide range of topics have demonstrated success in fostering competencies of FM residents [17, 18]. Short course or short intervention are other terms for an intervention of short duration—these terms are likely to be more common globally. The integration into an existing program (KWBW) is a specific feature of compact interventions according to the Heidelberg Model. For now, studies exploring the effects of compact or brief interventions on self-care in FM residents are scarce. In other specialties, such as pediatrics, there are studies of interventions that can be described as “compact” in scope and duration, but their results are inconsistent [19, 20]. Studies of interventions for residents in all medical specialties, regardless of their type or duration, widely report a positive outcome, suggesting that they contribute in some way to improving resident well-being [21]. Interestingly, there is no consensus on what the measurement of well-being in light of a preventive self-care intervention should be, as there is no widespread use of validated instruments. The very diversity of interventions leads to the difficulty of finding a common instrument or outcome [22], and it should be remembered that the target population of preventive interventions is not patients but medical colleagues who may not even experience stress at the moment of the intervention. Therefore, mixed methods were used to achieve the aims of this study. These were:

- to best characterize the multiple effects of a compact self-care intervention for FM residents,
- to specifically explore its behavioral changes in the short- and medium-term.

## Methods

### Design

The study examined the effects of a seminar in self-care for FM residents using a concurrent mixed methods approach. Mixed methods research designs are thought to gain a deeper, broader understanding of a certain phenomenon than studies that do not utilize the combination of a quantitative and qualitative approach [23]. The term “concurrent” relates to the timing of the two components. The authors performed a prospective, survey-based pretest/posttest interventional study (posttest 10–12 weeks after the intervention) with additional evaluation of a control group. However, FM residents who did not attend the intervention (= non-attendees) that made up the control group were assessed only once in a cross-sectional way. The second component of the study was an explorative qualitative approach, to better understand the different aspects of the impact of the compact intervention. Therefore, semi-structured interviews with the seminar attendees were conducted (almost at the same time, 12 – 14 weeks after the intervention). Both research components can be regarded as independent, i.e., the results from the post-survey (questionnaire) had no influence on the interview guideline (see Additional file 1).

### Sample/Setting

All FM residents registered on the KWBW Verbundweiterbildung^*PLUS*^© were offered voluntarily participation in a two-day seminar with a focus on self-care. A total of nine (*n* = 9) identical seminars (including the intervention) were offered from January to December 2018. The courses took place in three different seminar venues in Baden-Württemberg, Germany. FM residents were also invited to participate in the accompanying study (= intervention group, IG). FM residents in the study-team were excluded from study participation (*n* = 3). Those FM residents who did not attend a seminar (= non-attendees) were recruited by e-mail after the end of the intervention period (= control group, CG). Using this control group, all perspectives of FM residents should be captured to avoid bias. This approach has previously been described [18]. FM residents, who attended the seminar (= attendees) and participated in the study (= study participants), were recruited for interviews during follow-up.

### Ethics

The study was approved by the Ethics Committee of the University of Heidelberg (approval number S570/2015). Participation in the study was voluntary and not incentivized. All study participants provided signed informed consent.

### Intervention

An educational compact intervention on self-care (= seminar character) was developed. In 2018 this compact intervention (270 min.) was integrated into the annual two-day training session of the KWBW Verbundweiterbildung^*PLUS*^©. The target number of attendees was *n* = 25 FM residents per course. A detailed description of the seminar contents has already been published [18]. In a nutshell, the compact intervention was a tightly scheduled comprehensive training in self-care offering a broad spectrum of input and practical experience. Its main educational objective was to impart basic knowledge in practicing self-care. Further seminar goals were to provide basic knowledge about stress reactions, to empower the FM residents to identify their individual stressors, and to introduce different techniques for time management and relaxation. To achieve these goals, a variety of methods were used, ranging from plenary lectures and group discussions to practical exercises in imagination, Progressive Muscle Relaxation and a short meditation. The hidden curriculum aimed to induce a personal reflection and to establish an affirmative attitude towards self-care, being expressed with subsequent statements in the interview.

### Data collection

#### Quantitative data

FM residents willing to participate in the accompanying study were asked to complete a paper-based questionnaire right before the start of their individual seminar (T1). Ten weeks after the seminar (T2) they received an email invitation to complete an online questionnaire (SurveyMonkey, SurveyMonkey Europe UC, Dublin, Ireland). Methods for quantitative data collection were reported in detail [18]. Non-attendees of the seminar were still invited to participate in the study (CG). They were invited by e-mail to complete an online questionnaire (SurveyMonkey, SurveyMonkey Europe UC, Dublin, Ireland) in March 2019 but were obviously not interviewed for the purpose of this study. Data collection was completed in May 2019.

#### Qualitative data

The qualitative data used for this study was collected exclusively from seminar attendees who were willing to also participate in the study. First component of qualitative data was free-text sections at T2 (s. above). Additionally, attendees from any of the seminars were recruited for semi-structured telephone-interviews 12 to 14 weeks after the intervention (T3).

#### Integration of data

Qualitative data was partly used to support the quantitative findings and to complement their message (in terms of illustrative examples). For example, the change of behavior could be claimed in the questionnaires and examples of this were collected through the subsequent interviews. However, it is important to understand that the results from the questionnaire had no influence on the interview guideline, as qualitative and quantitative data collection occurred almost in parallel. For integration the two forms of data were analyzed separately and then merged in the discussion [24].

### Measures and outcomes (survey)

No suitable pre-existing instrument for evaluation of the compact intervention could be identified. Therefore, the study authors developed an assessment questionnaire, allowing tailored assessment of study outcomes [18]. The main outcomes of the quantitative part were to evaluate (I) self-rated change of cognition and (II) change in behavior. The aim of the qualitative part was to elucidate all possible effects of the compact intervention on study participants´ competencies (i.e., on their knowledge, skills and attitudes) as well as all sorts of induced behavioral changes (qualitative outcomes). Three versions of the questionnaire were developed by an interprofessional team and validated with think-aloud technique (assessments at T1, T2 and the CG).

For this study, all questionnaires included the same *n* = 5 sociodemographic items. At T2 as well as for the CG, the questionnaires included three statements about the cognition of risk:

- *“Physicians are at higher risk for depression and burnout than other professions.”*
- *“Family physicians are at higher risk for depression and burnout than other physicians.”*
- *“How important do you think educational courses on self-care are during postgraduate training?”* (Likert 1–5).

Only at T2 (and of course not for the CG) the questionnaire included three additional items: An evaluation of the intervention in terms of personal health effects of attendees (Likert 1–5, not all helpful – very helpful), and two yes/no-questions (whether attendees changed their behavior after the intervention + whether they shared the content of the intervention with others).

### Interviews

Interviews were performed as semi-structured telephone interviews by a specifically trained researcher with audio recording (MP3/Digital Recorder, SANGEAN). The interview guide (see Additional file 1) was developed in an interprofessional team (*n* = 4), whose members were familiar with the program, needs of the targeted learner-group and concepts of self-care. It was piloted using think-aloud-technique with two graduates from the program with minor revisions before use.

### Analysis

#### Surveys

All quantitative data was analyzed using the statistical program SPSS (IBM Statistics, Version 25). Characteristics of FM residents were summarized using descriptive statistics (category, frequency, mean with standard deviation, and median with interquartile range (continuous variables)). Chi-square tests were used to detect differences in frequencies between the groups and Mann–Whitney-U tests for differences in rank and continuous variables. Differences between T1 and T2 were analyzed using t-tests for dependent samples and McNemar-tests. Characteristics of study participants at T1 and T2 were compared to characteristics of participants who filled in the T1 questionnaire only using chi-square tests and t-tests for independent samples to analyze non-response bias. Free-text sections were screened and categorized according to Kuckartz [25] by two independent researchers experienced in qualitative analyses. Translation of qualitative data to English was validated by four independent researchers and a native English speaker.

#### Interviews

Interviews were transcribed verbatim (German). Data was analyzed by three different researchers using the structured qualitative content-analysis approach of Kuckartz [25] and with the aid of atlas-ti (ATLAS.ti Scientific Software Development GmbH, Berlin, Germany). Main categories were previously defined (pre-, peri-, and postinterventional aspects of the intervention, with peri-interventional referring to all content that first came to the participants' minds at the time of the intervention, as well as an evaluation of the seminar) whereas the subcategories and codes were inductively derived from the data. All quotations in the manuscript (incl. Table 2) were forward translated by the research team and critically reviewed and revised by a native English speaker (researcher, FP).

## Results

**Fig. 1:**
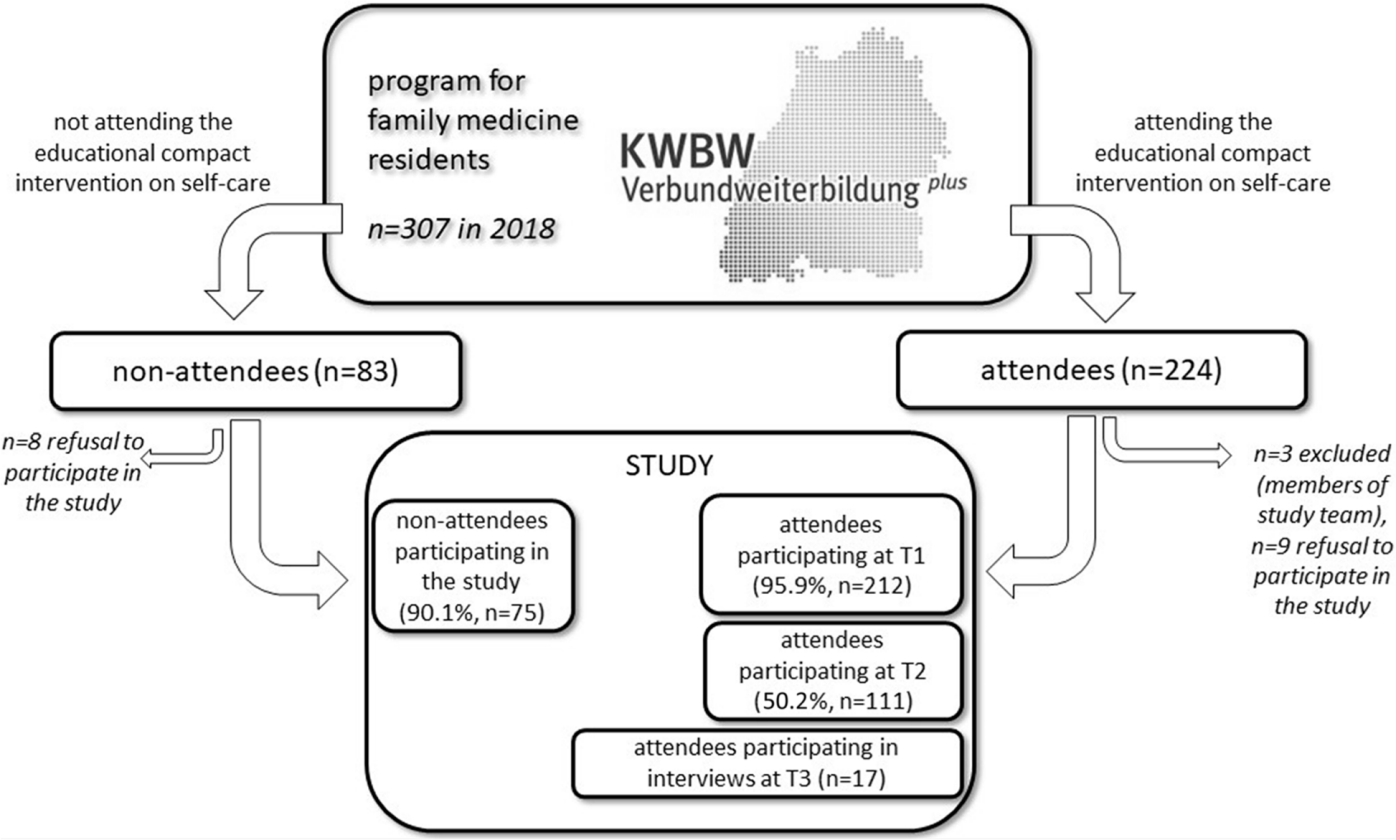
Flow chart illustrating the study´s steps and their sequence

The study took place in 2018: *n* = 401 FM residents were registered in the program, of which *n* = 307 generally participated (= active). Of these “active” FM residents, *n* = 224 attended one of nine independent two-day training programs (IG), all of which featured the self-care seminar (mean per course *n* = 25, range 21–31). Due to their involvement in the conception of the study, *n* = 3 FM residents were excluded, resulting in *n* = 221 attendees being potential study participants. 93.5% (*n* = 287) of active FM residents participated in the study (intervention group (IG): *n* = 212, control group (CG): *n* = 75). At T1, 212 pre-intervention questionnaires were returned (response rate: 95.9% from total of *n* = 221 attendees who could potentially have participated). At T2, 111 post-intervention questionnaires were completed (response rate: 50.2% from total of *n* = 221 attendees who could potentially have participated). Seventy-five FM residents participated in the CG (response rate derived from group of “active” residents: 90.1%). Seventeen FM residents were recruited for interviews (see Fig. 1).

### Sociodemographic data

Table 1 depicts participant sociodemographic data. A total of 94.1% (*n* = 16) of interviewees were in their fourth or fifth year of training.

**Table 1:** Sociodemographic data of family medicine residents (*n* = 287)

		**IG T1** (*n* = 212)	**IG T2** (*n* = 111)	**IG T3** (*n* = 17)	**CG** (*n* = 75)
**Gender**	Female	163 (76.9%)	84 (73.9%)	12 (70.6%)	52 (69.3%)
(n, %)	Male	48 (22.6%)	27 (22.5%)	5 (29.4%)	16 (21.3%)
	Unknown	1 (0,5%)	0 (3,6%)	0 (0.0%)	7 (9,3%)
**Age in years**	Md (Q1; Q3)	34 (32;39)	34 (31;37)	33 (31;36)	34 (32;40)
**Year of training**	Md (Q1; Q3)	4 (3;5)	4 (3;5)	4 (4;5)	5 (4;5)

*IG* intervention group, *CG* control group, *T1* before the intervention, *T2* 10-12 weeks after, *T3* interviews 12 – 14 weeks after, *p* p-value, *SD* Standard Deviation, *Md* Median, *Q1, Q3* interquartile range

### Quantitative data

#### Change of cognition

After the compact intervention, 56% of the FM residents rated the intervention to be very helpful (*n* = 24/111) or helpful (*n* = 39/111) for their actual personal well-being (mean 3.6, SD 1.0) whereas 15% rated the intervention as not helpful (*n* = 14/111) or not helpful at all (*n* = 2/111).

Risk awareness was similar in IG (at T2) and CG: IG rated the statement *“Physicians are at higher risk for depression and/or burnout than other professions”* with a mean of 3.92 (SD.93) on a 5-point Likert scale similar to the CG who rated it with 3.9 (SD 1.01). *“Family physicians are at higher risk for depression and burnout than other physicians”* was rated with a mean of 2.76 (SD. 88) in the IG and with 2.73 (SD 0.95) in the CG. Finally, *“How important do you consider educational courses on self-care during postgraduate training?”* was rated with a mean of 4.23 (SD 0.86) in the IG and with 4.03 (SD 0.90) in the CG.

#### Change of behavior

A total of 36% of participants at T2 (*n* = 40/111) declared that they had actively changed their behavior. At T2 71.4% (*n* = 35/49) of those who worried about negative health-effects due to their medical profession, chose to act. This was a significant increase of those willing to act in comparison to T1 (T1:T2 *p* = 0.01).

#### Transfer of competencies

Half of the participants had passed content of the course to others (*n* = 56/111). Findings from the free-text fields of the questionnaire on what was actually transferred to others can be found in the presentation of qualitative results below (see Table 3).

### Qualitative data

#### Qualitative data from free text sections

Measures taken due to the intervention are shown in Table 2.

**Table 2:** Behavioral changes of FM residents due to the intervention (*n* = 40/111)

Workplace	I appreciate breaks I have stopped going to work when I´m sick I have quit my current working position I now take the patient’s problems less personally
Familial	I am more responsive to the needs of my partner and my family
Relaxation	I have started doing nothing during free-time and enjoy it I sometimes ask myself how I feel and if I need a break I have enrolled in a relaxation course I do more meditation I schedule time for conscious relaxation I use imagination techniques I have implemented small exercises into everyday life
Physical activities (sports)	I do more sports I cycle to and from work I exercise more
Change of attitudes	I think positively more often I accept help I can put things to one side I am less of a perfectionist I want to give space for greater aspirations and nurturing the soul I have increased time for myself I actively use my resources I try to reach a better work-life balance
Time management	I have used the Pareto principle I have planned time for myself I have planned time for exercises
Others	I have started to pay attention to my problems I have increased my self-reflection I have been reminded of personal competencies I have started to enjoy my free-time I care for myself I have changed my diet and lost eight kilograms in weight

10-12 weeks after intervention, *FM* family medicine, qualitative content analysis of open text answers

Table 3 depicts which contents were passed on. Those contents can roughly be summarized as competencies in time-management, relaxation techniques as well as basic principles of stress and burnout syndrome and their prevention.

**Table 3:** Transfer of course-content by family medicine residents to third party members (*n* = 56/111)

Time-management	Relaxation techniques	others
- Time-management, not specified (21) - Dealing with procrastination (including Pomodoro technique) (7) - Avoidance of perfection including Pareto principle (4) - Optimization of operational sequences in the practice (3) - Prioritization including Eisenhower principle (3) - Parkinson’ law (1) - Attention curve over the course of the day (1) - Identification of time-thieves (1) - How to deal with e-mails (1) - ALPEN strategy for time-management (1)	- Relaxation techniques, not specified (9) - Mindfulness (8) - Progressive Muscle Relaxation (7) - Yoga (3) - Imagination techniques (2)	- Basics about stress and burnout, not specified (2) - Relevance and significance of psychosocial health (2) - Prophylaxis of burnout syndrome, not specified (1) - Improved awareness and understanding of stress and stressors (1) - Interindividual differences in stress and dealing with strains (1) - Learning to say no/ self-protection (1) - Change of cognition as part of self-care (1)

#### Interviews: categories

Twelve to fourteen weeks after the intervention, *n* = 17 interviews with FM residents who had been part of the compact intervention could successfully be performed. Data was categorized into two main categories: peri- and postinterventional aspects, with peri-interventional referring to all content that first came to the participants' minds at the time of the intervention, as well as an evaluation of the seminar. Tables 4 and 5 (i.e. part I and part II) summarize all codes whereas a coding-tree is presented in Table 1 (see Additional file 2).

**Table 4:** Part I: Mid-term (T3) review on an intervention in self-care by FM residents (*n* = 17)

Main category	Subcategory	Codes
**PERI-** **interventional**	Thoughts and feelings (during the course)	- Reflection on handling personal health - Reflection on personal stressors and experiences - Reflection on risks for physicians - Surprise about prevalence [of stress] amongst colleagues - Positive overall impression - Reaffirmation to act correctly - Relaxing effects due to practical exercises (PMR) - Decision for another way of managing personal health - Seminar important but too late (in their career) for personal development
	Strengths	- Educational concept - Gain in knowledge - Use of exercises - Induced self-reflection - Affirmation of self-care-oriented behavior - Problem-oriented approach - Evidence-based introduction on self-care including review of literature - Identification of personal behavior and individual stressors - Open learning-atmosphere enabling (confidential) discussion of individual stresses - Wake up call to act
	Weaknesses	- With regards to content, no weaknesses - Dysfunctional working conditions (swimming with sharks) was too short - Too short on time - Insufficient training in the practical exercises - Too much of information - Pace too fast
	Linkage to previous competencies	- Awareness of knowledge - Repetition of practical exercises - Repetition of methods
	Fun / learning atmosphere	- Interaction - Group cohesion - Practical exercises for relaxation techniques - Activation exercises (at the beginning)

*FM* family medicine, *T3* 12–14 weeks after the intervention, *PMR* Progressive Muscle Relaxation**.** Peri-interventional: all content that first came to the participants' minds at the time of the intervention, as well as an evaluation of the seminar

**Table 5:** Part II: Mid-term (T3) review on an intervention in self-care by FM residents (*n* = 17)

Main category	Subcategory	Codes
**POST-** **Interventional**	Thoughts and feelings during the interview (in review of the intervention)	Positive to look back on Useful [methods] for bad times Motivation boost Reaffirmation of personal behavior Perfectionism as an obstacle Call for self-care No personal need for self-care Implementation is difficult Too late for me, young doctors are the target group
	Mid-term benefits	Organization of clinical practice Practical exercises (PMR, Imagination) Time-management (Pomodoro, timetables) Prioritization (Pareto, Eisenhower matrix) Concept of mindfulness Awareness of stressors Understanding and acceptance of self-care Reaffirmation of a self-caring attitude Beneficial for patients to learn
	Changes of cognition and behavior	No change in attitude Help seeking Reduction in perfectionism Awareness of body signals Education for patients and myself Initiation of a self-caring attitude
	Implementation into daily life	Implementation does not work Concept of mindfulness Time-management in general Time-management during work Time-management in the family Relaxation techniques (PMR, Imagination) Reactivation of previous self-caring behavior Reactivation of life outside of work
	Transfer to others (to whom?)	Pomodoro-technique (spouse/partner) Pomodoro-technique (own children) Prioritization (spouse/partner) Concept of mindfulness (patient) Relaxation techniques (PMR, Imagination) (patient)

*FM* family medicine, *T3* 12–14 weeks after intervention, *PMR* Progressive Muscle Relaxation

#### In-depth interviews

##### Effects during the compact intervention

Peri-interventional codes, i.e., those referring to all content that first came to the participants' minds at the time of the intervention, as well as an evaluation of the seminar, were categorized into (1) thoughts and feeling, (2) strengths and (3) weaknesses, (4) linkage to previous competencies as well as (5) fun (learning atmosphere) during the intervention (Table 4). Participants described self-reflection of personal health during the course which could be accompanied with intense feelings:

Interviewer: “Can you recall what you thought and felt after the day? What were your impressions?”

#2: “Yes. Well, I remember that I ran down to the stream [outside the seminar´s venue] and sat down.“ (both laughing)

Interviewer: “Really? To let everything [the seminar content] sink in?” (laughing)

#2: “Exactly! (laughing). Because I thought that I need help!” (laughing)

Interviewer: “So, was it a bit too much information and therefore a bit overwhelming? Or would you say, of course not? “ (laughing, too)

#2: “No! I enjoyed it!”

An open and trustful learning atmosphere and the stimulus to act were named as strengths of the intervention. Asked for weaknesses of the intervention some interviewees declined to respond, whereas one missed debating about dysfunctional working conditions and another participant thought the course was rather tight in terms of time.

#11: “However, I was also thinking about it more deeply. “What could I do?”. A lot was obvious to me – I knew I needed to look after myself better, however hearing it again, particularly hearing the stories again, made me decide for myself that I really have to change something [in my life].”

The majority of interviewees could successfully link new and previous competencies, e.g. relaxation techniques or repeated realization of self-care. A positive and amusing learning atmosphere was achieved through the interactive conceptualization and active exercise of relaxation techniques.

##### Effects after the compact intervention

Post-interventional codes were categorized into (1) thoughts and feelings during the interview and in review of the intervention, (2) mid-term benefits, (3) changes of cognition and behavior as well as (4) implementation into daily life and (5) transfer to others (Table 5).

The majority of interviewees felt the seminar to be useful both, in general and for their actual well-being, were surprised by its effectiveness and regarded it as well as a reaffirmation of their efforts.

#11: “…And how through attending the course I learned, amongst other things, that these little steps can still have a big impact. You always think: “Yeah it’s obvious that I need to change the way I live”‚ however I don’t end up doing it because it ‘s too much hassle.“

One of the main mid-term benefits was the gain of competencies in time-management for daily work. Some participants mentioned that the course did not change their minds because they have already had a positive and self-care-oriented point of view, whereas others said the intervention has induced a self-care-oriented attitude, which had previously been some kind of taboo to them:

#11: “Exactly, [perfectionism] is a main threat, however what I have really learned, is that the thing I recommend to patients – to look after themselves – seems to be right.”

Regular implementation of course content after the intervention was described as challenging by *n* = 7 interviewees. Many had successfully implemented course content, especially time-management skills including daily schedules or avoidance of over-time work and regular practice of relaxation techniques. Some participants mentioned that they had reactivated regular sports and active recreational activities due to the intervention. In summary, behavioral change was reported by many interviewees:

#11: “Yes! I go running at least twice a week. I also use the imagination techniques we learned on the course almost every evening. And at the end of the day, I look back at the day and I am mindful about my breathing. I must say that I have gained incredibly from that. “

Interviewer: “So, would you say that you now go running and do the relaxation exercises as a result of the course’s impact on you?

#11: “Yes! “

Various study participants mentioned that they have passed course content to others, mostly within their family or towards friends but also to their patients. The transferred content included competencies in time-management as well as relaxation techniques and mindfulness.

#15: “Ok, yes it was this Pomodoro-method. […] I found this very good and helpful. I’ve even taught it to my son for his learning-stress, and he’s learned it according to the method. Actually, I (as an “old-timer”) have learned something new that I can use in daily practice.”

## Discussion

To the best of the author’s knowledge this is the first study which explored behavioral changes after an intervention in self-care among FM residents in Germany. The study demonstrates that even a brief intervention of 270 min has the power to foster FM residents' willingness to act in a self-care manner, induce behavioral change, and support the transfer of self-care skills to others. Interviewing seminar attendees helped to understand that the compact intervention is effective by a low-threshold well-balanced composition of information, self-reflection and motivation in a positive, trustful and even amusing learning atmosphere which is supportive and gently integrates FM residents’ needs and previous competencies. Generally, studies analyzing behavioral changes following educational interventions in medicine are scarce and no study could be found which investigated effects of a comparably short educational intervention in self-care among residents. This is not overly surprising as it is challenging to rate the success of such an educational intervention as subsequent changes in standardized psychometric tools appear unlikely, even if there are isolated reports about such effects: Back in 1991 a stress management workshop was shown to lead to significant short-term improvement in stress and Maslach Burnout Inventory (MBI) test scores for medicine and pediatrics residents [19]. However, twenty years later Martins et al. reported that a brief intervention for pediatric residents failed to be effective in reducing burnout prevalence measured by the MBI in a pre-post design [20].

Regardless of these inconsistent study results, the intervention studied in this paper was never developed to "cure" FM residents suffering from burnout syndrome, but to offer prevention by promoting self-care techniques to the broad mass of FM residents (representing a spectrum from very stressed and burdened to subjective well-being), to raise awareness for the topic of self-care, and to show ways of self-care. For now, no valuable surrogates have been described for this intervention purpose. This is why integrated a qualitative, explorative approach. Below, the authors discuss whether behavior change (including transfer to others) indicate sustained gains in competence.

### Change of behavior

In a longitudinal randomized controlled study on a training program for medical residents a statistically significant increase in self-efficacy and decrease in stress to communicate with their patients was reported, although no changes were noted in burnout [26]. Therefore, the study established, in very basic terms, a link between a person-centered intervention and certain behavioral changes in residents.

Behavior change is an effect that should also be focused on in this study. After the intervention, FM residents were more willing to take care of their own health. Change of behavior is described in five stages [27]. "Precontemplation", a phase were individuals are unaware of their risks, is followed by a phase in which people are aware that a serious problem or a risk exists and are thinking about overcoming it ("Contemplation"). More than half of the participants rated the intervention to be helpful or very helpful for their actual personal well-being. This gain in well-being might be due to the opportunity to care for one-self during the course. The power to change attitudes by an educational compact intervention has been observed in a previous study of the authors [17]. The data in this study suggest, that participants as well as non-participants already had a broad awareness and thereby the majority entered the intervention in a phase of contemplation. It appears reasonable that one main effect of the compact intervention on self-care was to empower the FM residents to care for themselves (involving stages 3–5): In a third stage ("Preparation") individuals begin planning the change they want to make. This is followed by the individual implementing the first changes into daily life ("Action"). The last stage ("Maintenance") concerns preventing relapse and consolidating the gains from the Action-stage. According to Lally et al., it takes 18—254 days for a person to form a new habit – this equates to 66 days on average for a new behavior to become automatic [28]. Participants demonstrated that they took care of themselves after the intervention, e.g., not going to work when feeling sick, and stated that they changed their daily routines. Quite a few described re-activation of previous skills and re-affirmation of a self-care-oriented behavior. Four out of five stages from Prochaska and DiClemente’s theory on habit formation could be observed by the time of the interview.

Finally, residents have also passed on knowledge and skills to others who had not attended the seminar. The outcome of the transfer of competencies was not expected beforehand – it is an additional gain of knowledge that resulted from the interviews. Given the well-described "learning-by-teaching benefit", it may have been these attendees who benefited from the seminar in a particularly sustainable way [29], as teaching can be regarded as the facilitation of learning [30]. These “teachers” of self-care have apparently started to maintain their competencies (stage 5 in Prochaska and DiClemente’s stages of change model).

### Mechanisms of educational compact interventions

An educational compact intervention on self-care has not only the power to foster knowledge, skills and attitudes, but also enhances personal health and induces behavioral changes. This is somehow remarkable because of its brevity (= 270 min). The strengths of the intervention included an interactive and problem-oriented approach with practical exercises designed to connect to the learners' previous experiences. Most valuable was the open and positive learning atmosphere, which the authors assume generated positive feelings in the attendees.

However, the positive atmosphere was supported by other characteristics of the seminar: First of at all, the KWBW Verbundweiterbildung^*PLUS*^© is a voluntary program. Secondly, attendees have previously reported that the main reasons for participating in the curriculum are 1) the learner-oriented, interactive and case-based seminars with FM specialists as moderators of competencies and 2) the exchange with other FM residents, positive atmosphere and the encouraging stimulus to proceed with their careers in FM [31]. Participation in the seminar-program is described as a boost in motivation [31]. Therefore, the educational compact intervention also benefited from a strong group cohesiveness as well as a positive and supportive learning atmosphere.

Altogether, the educational compact intervention in self-care induced an encouraging impetus to act and facilitated a behavioral change. Furthermore, educational compact interventions have been shown to increase knowledge, skills and attitudes. However, they appear to require embedment within a program with a strong group cohesiveness. They have not been shown to be successful in e-learning. Finally, long-term effects need to be investigated.

### Limitations

A thorough psychological assessment of the FM residents before and after the intervention would have further strengthened the study. However, for reasons of confidentiality, the authors decided not to conduct a pre-post intervention with individual-level psychometric assessments. Instead, the authors published an anonymously conducted cross-sectional analysis of psychological distress within the target group [32]. Since the authors of this study could not find a tool that would have satisfactorily addressed all study goals, they developed the questionnaire themselves. It can be considered a weakness of the study that ex-ante reliability and validity testing of this targeted tool could not be performed due to time constraints. In addition, questionnaires always involve the risk that, unlike in an interview, it is not possible to respond to the given answers. Misunderstandings on the part of the study participants may also occur more frequently in this way. Accordingly, one participant mentioned yoga as course content, but there were no (practical) yoga exercises. However, to counterbalance this weakness, the authors used a mixed-methods approach including interviews.

Finally, the study only records medium-term effects. This does not allow a valid conclusion to be drawn about possible long-term effects in terms of permanent changes in behavior, although permanent retention of a new behavior can be assumed after 66 days [28].

## Conclusions

An educational compact intervention in self-care has the power to foster FM residents' willingness to act in a self-care manner and induce behavioral change. What was additionally demonstrated by the qualitative part of this mixed-methods study, although this was not expected as an outcome: The intervention could also support the transfer of self-care skills to patients, colleagues, or family members. FM residents were successfully empowered to integrate self-care in their daily life, through the active execution of beneficial behavior to endure external strains and by sensitization of their professional and home environments. In doing so, FM residents developed strategies to sustain their personal health and thus their ability to work sustainably as a physician. This is not only crucial for their colleagues and patients, but also for the healthcare system as a whole. Further studies should investigate possible long-term effects of similar compact interventions in self-care.

## Supplementary Information

**Additional file 1. **Interview guide.

**Additional file 2.** Table 1 (coding tree).

## Data Availability

The datasets generated during and analyzed during the current study are not publicly available due to the lack of an adequate IT solution for this purpose but are available from the corresponding author on reasonable request.

## Abbreviations

CG: Control group
FM: Family medicine
FPs: Family physicians
IG: Intervention group
KWBW: Kompetenzzentrum Weiterbildung Baden-Württemberg
MBI: Maslach Burnout Inventory
PMR: Progressive Muscle Relaxation
SD: Standard deviation
T1, T2, T3: Different time points
Tab: Table
