# Supplementary material for: Effects of an educational compact intervention in self-care – a mixed methods study with postgraduate trainees in primary care
Source: BMC Prim Care. 2023 Jun 16;24:124. doi: 10.1186/s12875-023-02074-w (PMC10273587; doi:10.1186/s12875-023-02074-w)
Supplement: Supplementary file 1 — Additional file 1. Interview guide. [file 12875_2023_2074_MOESM1_ESM.docx]

**Interview guide** (Cave: translation for international readers – original version in German language!)

Date / Time / No.

**Thank you for agreeing to participate in this interview! As written in the email, our concern is to continuously improve the Verbundweiterbildung*^PLUS^*© program. We need 20-30 minutes and will discuss the seminar [...].**

**It is very important for me to know your honest opinion / PAUSE / First of all, I would need some data from you…** **But do you have another question that we should clarify in advance? /**

**Ok then…**

| Sex | Age | Year of training | Inpatient or outpatient training | Year of entry into the Verbundweiterbildung*^PLUS^*© program |
| --- | --- | --- | --- | --- |
|  |  |  |  |  |

***I would start the recording now, ok? -> ON***

1. **Again, to make sure, your consent for recording has been given?**
2. **... and you have participated in the two-day seminar of the Verbundweiter-bildung*^PLUS^*© program with focus on physician self-care?**
3. **Did you also participate in the follow-up evaluation?**

**Good, here we go....**

1. **Why did you sign up for this two-day seminar?**

(Was there a reason, for example, the neurology seminar? Or the seminar on physician self-care?)

1. **Did you have expectations?**

*Or:* **Did your expectations come true?**

(*Or:* Did you have specific expectations about the friday seminar on physician self-care?)

**What did you think and feel (immediately) after the two-day seminar?**

Which of these thoughts were related/not related to the seminar?

1. **What do you think and feel about the seminar on physician self-care *today*?**
2. **What did you enjoy the most?** What did you not enjoy at all?
3. **Which seminar contents have been helpful from your current point of view?**

*Or:* **What has brought you further in retrospect?**

Why did this in particular help you?

What was so special about it?

I didn't understand that – what exactly was helpful?

What would have brought you further?

1. **What did you change in your life because of the seminar on physician self-care?** Or did you integrate anything from the seminar into your everyday life? If so, what was it?
2. **(Do you have any suggestions for changes to the seminar on physician self-care?)**
3. **What is your conclusion from the seminar on physician self-care?**

***CONCLUSION***

1. **Did we forget anything else? Is there anything that is still on your mind?**

***OFF***

**Thank you for your participation!**
