# Supplementary material for: Effects of an educational compact intervention in self-care – a mixed methods study with postgraduate trainees in primary care
Source: BMC Prim Care. 2023 Jun 16;24:124. doi: 10.1186/s12875-023-02074-w (PMC10273587; doi:10.1186/s12875-023-02074-w)
Supplement: Supplementary file 2 — Additional file 2. Table 1 (coding tree). [file 12875_2023_2074_MOESM2_ESM.docx]

| **Tab. 1 (appendix)– Coding tree of qualitative analysis of interviews with FM residents after an educational compact intervention on self-care (n=17)** | | | |
| --- | --- | --- | --- |
| **main** | **definition** | **example** | **coding** |
| **PERI-**  **interventional** | All content that first came to the participants' minds at the time of the intervention, as well as an evaluation of the seminar. | Thoughts and feelings during the course, strengths and weaknesses of the intervention as well as aspects of education as learning atmosphere and constructive alignment were coded in this category. | ***#15****: “Well, straight after I was very positive. I immediately went to the lecturer and told him I enjoyed how he delivered the course (…) that I didn’t have many expectations but that it was enjoyable, and have been delivered well. I was positively surprised and left in a good mood.”* |
|  |  |  | ***#2****: “In the end, everyone is in the same situation. but somehow you don't have to worry about it... I'll say... asking stupid questions. Yes - I always found (the learning atmosphere) quite pleasant...”* |
|  |  |  | ***#1****: “Well, I really liked that it was very interactive [child crying], especially about time-management and health behavior which was very interactive – also with the [relaxation exercise], which was demonstrated by the lecturer.* |
| **POST-**  **Interventional** | All contents that deal with aspects in mid-term review of the intervention. * | Thoughts and feelings during the interview in review of the intervention and gain of competencies retrospectively as well as mid-term effects like change of attitudes, implementation of behavioral changes and transfer to others were coded in this category. | ***#14****: “Finally, I really think that self-care is a very important topic. Because every one of us has to think about maintaining his workforce. And every one of us – whether a physician or not – only has limited energy. And this, naturally, has to reasonably be invested on the one hand – keyword time-management. But, then also to recognize when I am burdened, when do I need a break for myself and how can I manage a break? And this is why raising awareness and the recognition of risk becomes an important and relevant topic, because we as physicians are so keen to care for our patients but not for ourselves. And this is why I definitely think that such course on self-care makes sense – also in postgraduate medical education.”* |
|  |  |  | ***#18****: “What made an impression on me - it was that someone did not care about punctuation or use of upper and lower case in his e-mails. Because I am someone, who triple-checks everything. I continue to re-check my e-mails, however now care less when sending text-messages. I just want the other one to understand what I am trying to say. It’s not an official document, no one will give it a second look. Not to overdo it and to lose the time forever. Or what I have also recognized, what my children taught me: to use voice assistance while writing text messages with my mobile phone or e-mails* |

**Note.** FM = family medicine, Main = main category, * Interviews were performed 12 to 14 weeks after the intervention
